# Case report of a ruptured right atrial aneurysm in a child and a comprehensive literature review

**DOI:** 10.3389/fped.2024.1369345

**Published:** 2024-04-02

**Authors:** Jie Zhou, Hao Xin, Qiao Li, Mei Zhu

**Affiliations:** Department of Ultrasound, Shandong Provincial Hospital Affiliated to Shandong First Medical University, Jinan, Shandong, China

**Keywords:** atrial aneurysm, echocardiography, pericardial effusion, right atrium, rupture

## Abstract

**Background:**

The right atrial aneurysm is a rare cardiac malformation of unknown origin. It is typically asymptomatic but can occasionally lead to life-threatening and serious complications.

**Case description:**

We present a case of a right atrial aneurysm in an eight-year-old child who experienced complications including rupture of the atrial aneurysm, thrombosis, and recurrent large pericardial effusions over a one-month period. Following surgical treatment, the child had a favorable prognosis.

**Conclusion:**

A congenital right atrial aneurysm may manifest as either a widespread enlargement of the right atrium or a localized, smaller sac-like protrusion. In the latter case, diagnosis can be challenging to confirm through transthoracic echocardiography alone, and may require a cardiac computed tomography angiography examination for a definitive diagnosis. For patients experiencing recurrent large volumes of bloody pericardial effusion within one month, and exhibiting no atrial enlargement but showing abnormalities of the atrial wall in echocardiography, it is important to be vigilant about the potential for atrial aneurysm rupture in the heart. Timely treatment is essential to prevent the progression of the condition, which could otherwise result in a poor prognosis.

## Introduction

The right atrial aneurysm (RAA) represents an uncommon cardiac anomaly, manifesting as either a congenital or acquired malformation. Typically of congenital origin, its onset can transpire at any stage of life, with a predilection for affecting both genders in equivalent proportions (1). It is noteworthy that almost all previously reported cases involved congenital giant RAA from fetus to adult. In our case, the RAA was smaller, and the patient presented with different clinical manifestations compared to previously reported cases. These included complaints of gastrointestinal symptoms and the absence of significant enlargement of the right atrium and cardiac arrhythmias. However, most importantly, this case presented with the complication of both atrial rupture and thrombosis in atrial aneurysm, which has never been reported previously and is extremely rare. Therefore, we decided to report this case in order to provide a reference for clinical diagnosis and treatment. The optimal treatment for RAA remains controversial. Currently, surgical treatment is the primary recommendation. Instances documented in literature propose that the resection of an RAA may serve as an efficacious curative surgical intervention (2). In clinical practice, it is important to develop an appropriate individualized treatment plan based on the patient's specific situation.

## Case description

An eight-year-old female presented to our emergency department exhibiting recurrent episodes of vomiting and abdominal distension persisting for a duration exceeding one month. Her condition was characterized by frequent and prolonged vomiting episodes, occurring approximately half of the day. She was occasionally accompanied by dizziness and weakness. Physical Examination: The patient exhibited a temperature of 36.8°C, a pulse rate of 154 beats/min, a respiration rate of 27 breaths/min, and a blood pressure of 85/69 mmHg. Although displaying cognitive clarity, the patient demonstrated reduced overall energy levels and presented with pallor evident in both facial complexion and conjunctival regions. Breath sounds in both lung fields were noted to be coarse, with a corresponding reduction in apical pulsation. Additionally, an observable enlargement was detected along the border of cardiac dullness. Cardiac auscultation revealed rhythmic and distant heart sounds, with no audible murmurs in the auscultatory areas corresponding to each valve. It is worth noting that the child had a history of external chest injury after falling two months ago. the patient had undergone treatment at another medical facility one month prior, during which pericardial effusion had been diagnosed without a definitive etiological explanation. During treatment at another hospital the child had elevated transaminases and liver function improved after hepatoprotective therapy was given. In addition, no substantial lesions in the digestive system were found by relevant laboratory tests, abdominal ultrasound and abdominal contrast-enhanced CT.

**Figure 1:**
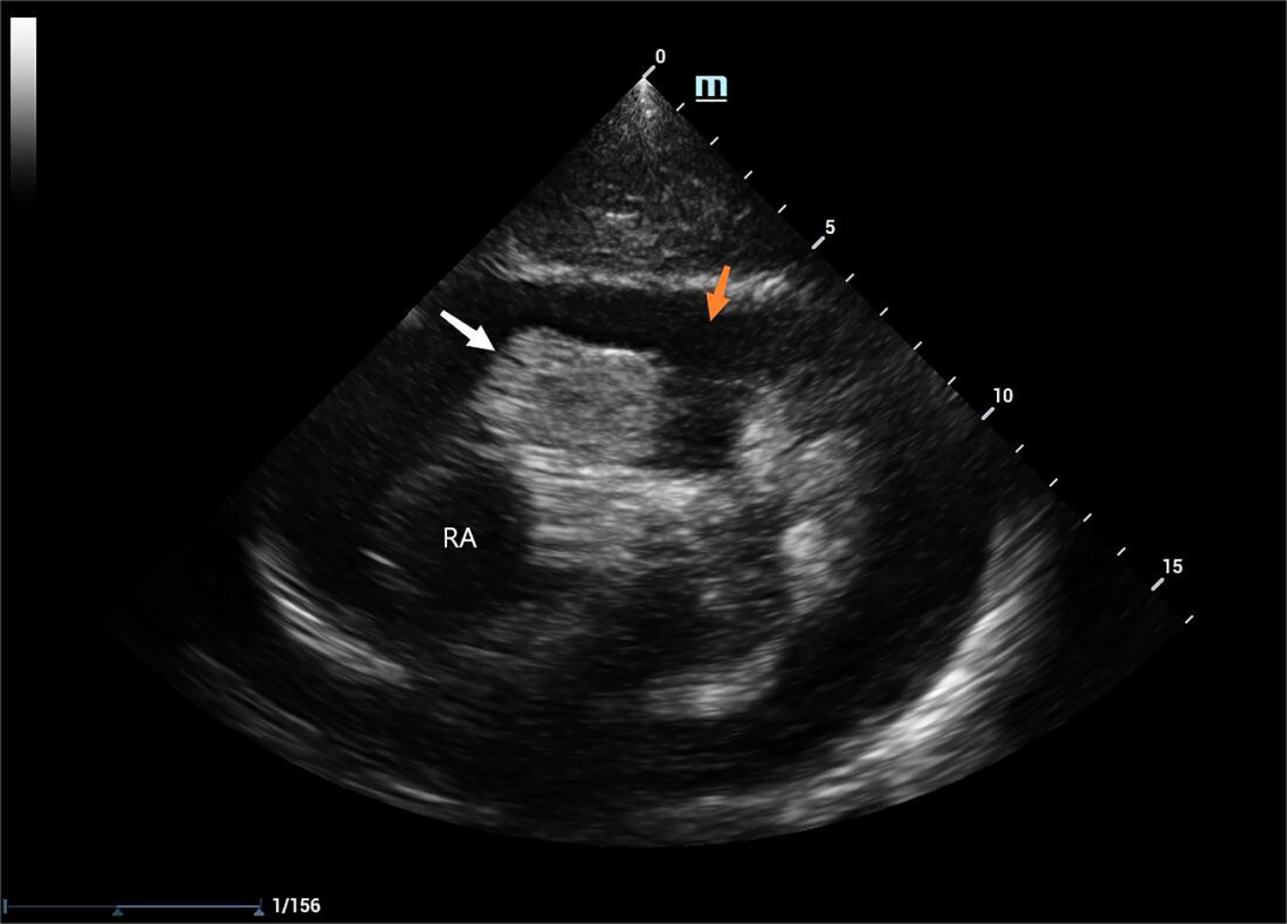
2D TTE: pericardial effusion (indicated by the orange arrow), RAA (indicated by the white arrow), RA: the right atrium.

Laboratory tests revealed a pro-brain natriuretic peptide level of 317 pg/ml, D-dimer level of 6.12 mg/L, and hemoglobin level of 81 g/l. The cardiac ultrasound examination disclosed a substantial accumulation of pericardial effusion characterized by fibrous streak-like echoes and large patchy hyperechoic echoes detected within the effusion (Figure 1). After draining the pericardial effusion, the cardiac ultrasound revealed hypoechoic exudate accumulation in the pericardial cavity and cystic solid echoes in the right auriculoventricular channel.

Examination of the pericardial effusion indicated turbidity and a reddish hue, indicative of an elevated concentration of red blood cells. No cancer cells, bacteria, or fungi were detected. Several other etiological tests were negative and anti-neutrophil cytoplasmic antibody (ANCA) antibody profile test was also negative.

**Figure 2:**
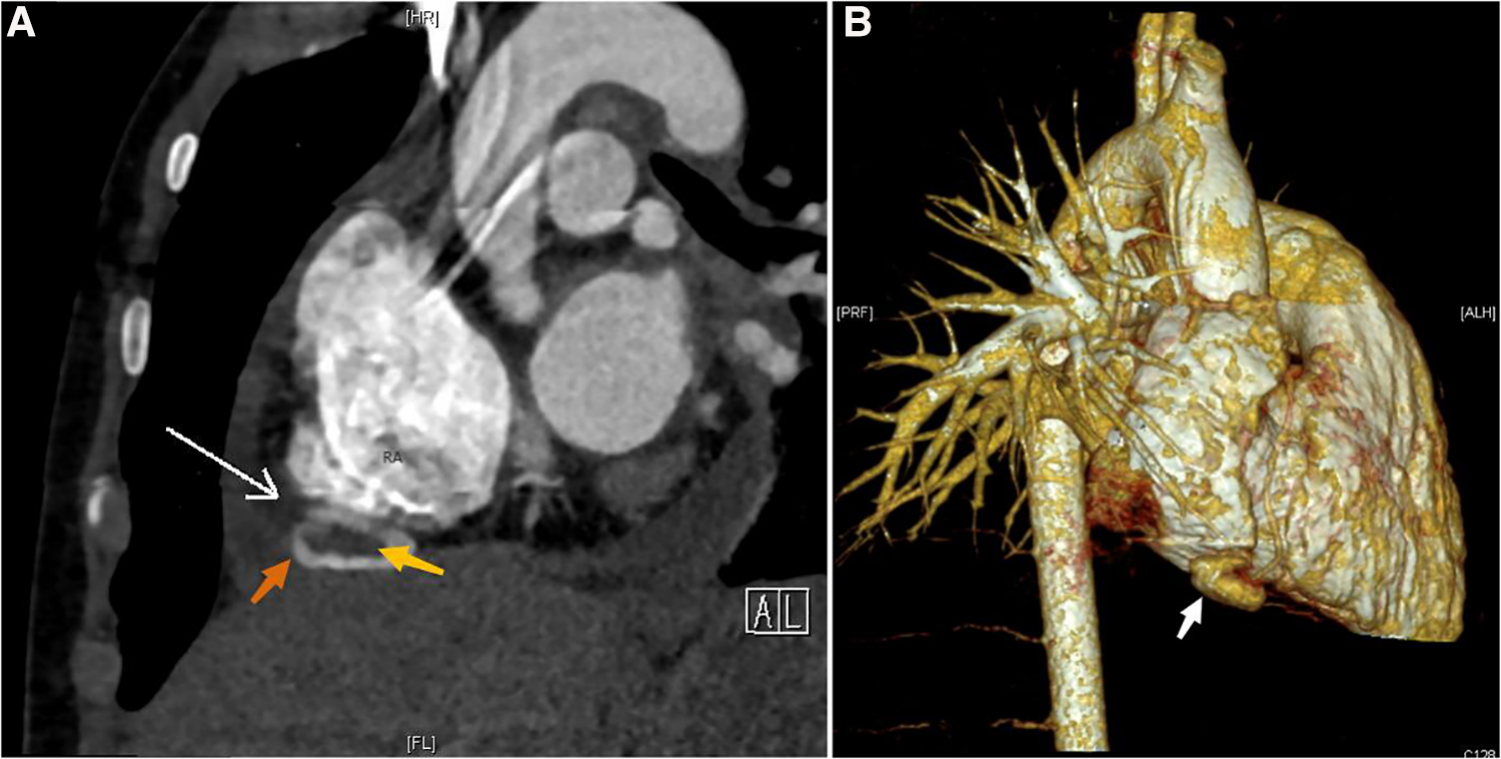
CTA and CT 3D reconstruction: (**A**) RAA (indicated by the orange arrow), RAA tumor neck (indicated by the white arrow), thrombus (indicated by the yellow arrow); (**B**) RAA (indicated by the white arrow).

Following an extensive analysis to rule out the possibility of neoplastic, inflammatory, and immune pericardial effusion, we highly suspected a rupture in the child's heart at this point and strongly recommended cardiac computed tomography angiography (CTA). The cardiac macrovascular CTA revealed a saccular pocket-like protrusion with hypodense foci inside at the lower margin of the right atrial atrioventricular sulcus. An RAA with thrombosis was not ruled out (Figure 2), along with bilateral pneumonic changes. This finding confirmed our suspicions.

**Figure 3:**
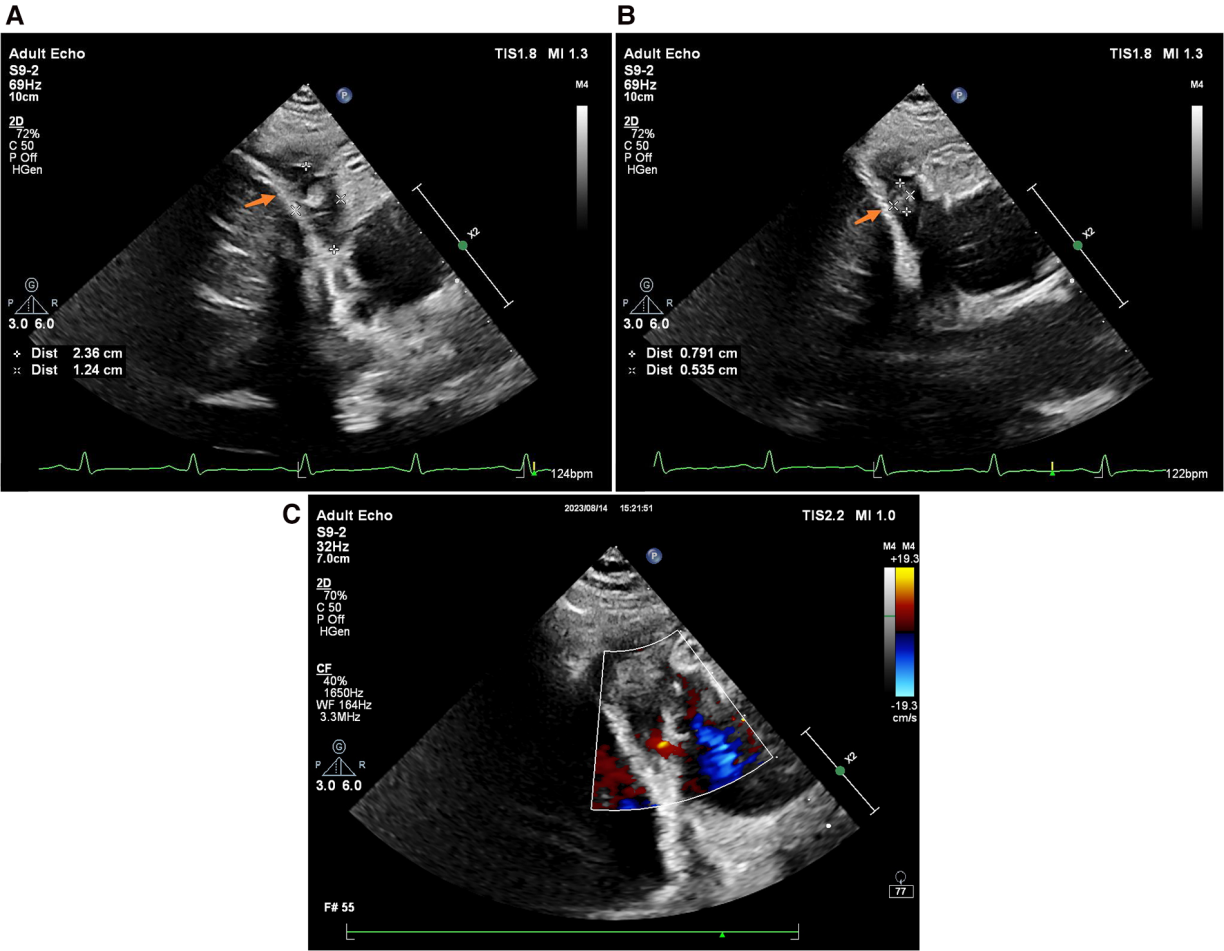
2D TTE (without pericardial effusion): (**A**) RAA (indicated by the orange arrow); (**B**) thrombus (indicated by the orange arrow); (**C**) color Doppler ultrasound.

A follow-up cardiac ultrasound revealed a hypoechoic mass detected in the right atrium and right atrioventricular sulcus, which was soft and measured approximately 2.36 × 1.24 cm, and a subrounded moderate intensity mass echo was detected within it, which was reduced in size compared to the previous one and measured approximately 0.7 × 0.5 cm (Figure 3). A small amount of fluid sonolucent area was detected in the remaining pericardial cavity, and behind the posterior wall of the left ventricle was approximately 0.45 cm deep, with localized thickening and adhesion of the pericardial visceral layer and wall layer, and areas of anechoicity could be detected.

Ultrasound examination led to the diagnosis of a ruptured RAA accompanied by thrombosis. A resection of the atrial aneurysm and atrial thrombectomy was performed under cardiopulmonary bypass under general anesthesia, and the intraoperative findings confirmed the diagnosis of echocardiography. Intraoperative exploration showed active bleeding from the posterior lateral aspect of the right atrial wall near the atrioventricular groove, the wall of the atrial aneurysm was very thin and ruptured, and the thrombus was cleared, and the neck of the atrial aneurysm was seen to be connected to the right atrial wall, with a rupture size of about 5 mm, which bled with the heartbeat, and with the surrounding wall of the atrium being weak. Continuous suture of the rupture and the surrounding weak atrial wall tissue. The postoperative pathological diagnosis confirmed the presence of a right atrial thrombus. The submitted sample exhibited thrombus tissue characterized by substantial inflammatory cell infiltration, exudation, and necrosis. The postoperative recovery was uneventful, with improvement in secondary symptoms of pneumonia and anemia. The patient was discharged following stabilization.

## Discussion

RAA is a rare congenital or acquired cardiac malformation of unknown etiology. True RAA was first described in 1968 by Morrow and Behrendt (3). It may occur at any age, from fetal development to adulthood (4). However, the majority of cases are typically identified in adulthood (1). Atrial aneurysm and diverticulum both manifest as aneurysm-like protrusions within the normal atria. However, their differentiation relies on distinct anatomical structures and pathological histology. The majority of individuals with RAA are asymptomatic, with incidental cardiac enlargement being identified during routine physical examinations. In cases where symptoms are present, palpitations emerge as the most prevalent clinical manifestation. Without timely intervention, serious complications such as tachyarrhythmias, thrombosis, pulmonary embolism, and right atrial rupture may occur, with supraventricular arrhythmias being the most common complication (1). In patients with a giant RAA, it is important to carefully differentiate it from Ebstein malformation, pericardial effusion, pericardial cysts, and pericardial tumors. The cause of congenital or acquired RAA is unknown. It is widely believed that congenital RAA is associated with localized congenital developmental abnormalities of the right atrial wall. Two primary hypotheses have been proposed. The initial hypothesis posits that the susceptibility of the right atrium to dilation, even under low pressure, may be attributed to abnormal intrinsic structural proteins such as collagen or pectinate muscles (5, 6). The second hypothesis implicates a congenital absence of myoblasts, potentially linked to viral infections or other injuries leading to embryonic muscle loss (7). In the case of acquired RAA, it is hypothesized that elevated right atrial pressure and enlarged right atrial volume are caused by the long-term effects of conditions such as pulmonary hypertension, congenital heart disease, or heart failure (8). RAA is generally of congenital origin, and in this particular case, the child did not exhibit any predisposing conditions, prompting consideration of a congenital etiology. Regarding the cause of the rupture, considering the child's previous chest trauma from a fall two months ago and a pericardial effusion one month ago, in view of this chronological relationship and in conjunction with the relevant laboratory tests, we believe that the rupture of the atrial aneurysm in this case was more likely to have been triggered by trauma.

Transthoracic echocardiography (TTE) is typically the preferred test for diagnosing RAA, and the diagnosis can be confirmed in most cases (9). However, in instances where confirmation of the diagnosis is challenging through TTE, a combination of cardiac CTA proves valuable in elucidating the diagnosis, as observed in this case. A distinctive feature of this case is the localization of RAA at the right atrioventricular channel, with no notable dilation observed in the right atrium. Instead, small cystic solid echoes were identified, localized within the right atrial wall. These echoes were challenging to detect without fluid lining in conventional TTE. The presence of an abnormality in the right atrial wall can only be inferred from the patient's clinical presentation and history, making it extremely difficult to make an accurate diagnosis based solely on cardiac ultrasound. Besides, the patient presented with abdominal distension, nausea, and vomiting, which are non-specific manifestations of cardiovascular system disease. Finally, this case presented complications of both atrial rupture and thrombosis in atrial aneurysm, which have not been reported previously and are extremely rare. The occurrence of this case enhances our understanding of this disease.

In conclusion, the accurate diagnosis of a ruptured RAA with thrombosis can be achieved through a comprehensive approach involving TTE, cardiac CTA, and a thorough examination of the patient's medical history. In patients presenting with recurrent substantial bloody pericardial effusion as a predominant symptom within one month, coupled with persistent low hemoglobin, elevated D-dimer levels, and absence of atrial enlargement but the detection of abnormalities in the atrial wall through TTE, heightened awareness should be maintained regarding the potential occurrence of cardiac atrial aneurysm rupture. Early surgical intervention in children with ruptured RAA combined with thrombosis can lead to a favorable prognosis.

## Data Availability

The original contributions presented in the study are included in the article/Supplementary Material, further inquiries can be directed to the corresponding author.
